# Cerebral oxygen saturation and peripheral perfusion in the extremely premature infant with intraventricular and/or pulmonary haemorrhage early in life

**DOI:** 10.1038/s41598-018-24836-8

**Published:** 2018-04-25

**Authors:** Thierry P. Beausoleil, Marie Janaillac, Keith J. Barrington, Anie Lapointe, Mathieu Dehaes

**Affiliations:** 1 Institute of Biomedical Engineering, University of Montréal, Montréal, Canada; 2 Research Centre, CHU Sainte-Justine, Montréal, Canada; 3 Department of Pediatrics, Division of Neonatology, CHU Sainte-Justine and University of Montréal, Montréal, Canada; 4 Department of Radiology, Radio-oncology and Nuclear Medicine, University of Montréal, Montréal, Canada

## Abstract

Extremely preterm infants are at higher risk of pulmonary (*PH*) and intraventricular (IVH) haemorrhage during the transitioning physiology due to immature cardiovascular system. Monitoring of haemodynamics can detect early abnormal circulation that may lead to these complications. We described time-frequency relationships between near infrared spectroscopy (NIRS) cerebral regional haemoglobin oxygen saturation (*CrSO*_2_) and preductal peripheral perfusion index (*PI*), capillary oxygen saturation (*SpO*_2_) and heart rate (*HR*) in extremely preterm infants in the first 72 h of life. Patients were sub-grouped in infants with PH and/or IVH (*N*_*H*_ = 8) and healthy controls (*N*_*C*_ = 11). Data were decomposed in wavelets allowing the analysis of localized variations of power. This approach allowed to quantify the percentage of time of significant cross-correlation, semblance, gain (transfer function) and coherence between signals. Ultra-low frequencies (<0.28 mHz) were analyzed as slow and prolonged periods of impaired circulation are considered more detrimental than transient fluctuations. Cross-correlation between *CrSO*_2_ and oximetry (*PI*, *SpO*_2_ and *HR*) as well as in-phase semblance and gain between *CrSO*_2_ and *HR* were significantly lower while anti-phase semblance between *CrSO*_2_ and *HR* was significantly higher in PH-IVH infants compared to controls. These differences may reflect haemodynamic instability associated with cerebrovascular autoregulation and hemorrhagic complications observed during the transitioning physiology.

## Introduction

Extremely premature infants born <28 weeks of gestational age (GA) are at higher risk of haemodynamic instability after birth due to their immature cardiovascular system and transitioning circulatory physiology^1^. Normal birth is marked by a decrease in pulmonary vascular resistance following lung expansion and by an increased in systemic vascular resistance due to placental removal and transition from fetal to neonatal circulation^2^. In the extremely preterm infant, this transition differs due to several factors. Particularly, the cardiovascular system is immature and the specific maturational processes are not completed. The immature left ventricle has to face an increased afterload following the interruption of fetal circulation^3,4^. These physiological changes increase the risk of hypotension and low systemic blood flow, which may lead to insufficient oxygen delivery and tissue oxygenation^5^. These alterations in haemodynamics early after birth may result in complications often associated with extreme prematurity in the first days of life, i.e. cerebral intraventricular (IVH) and pulmonary (PH) haemorrhages.

IVH occurs in about 30% of preterm infants born ≤28 weeks of gestation^6^ and PH, which refers to hemorrhagic pulmonary edema^7^, has an incidence of around 10% in the same population^6^. These haemorrhagic complications occur mainly in the first 72 hours of life and are associated with a wide spectrum of short- and long-term adverse outcomes. In particular, severe IVH can lead to post-haemorrhagic ventricular dilatation and/or white matter injury^8,9^. Severe IVH has also been related to cerebral palsy, epilepsy, learning disability, and visual and hearing impairments^8,10^. On the other hand, PH is often associated with severe respiratory distress syndrome^11^, hypotension and neonatal death in the acute phase, as well as severe bronchopulmonary dysplasia and reduced pulmonary function in the long-term^12^. Neonates who develop either or both IVH and PH have been reported to have more sensory and motor problems as well as cognitive impairments compared to healthy or term infants^4,12^.

Despite recent advances in monitoring techniques used in the neonatal intensive care unit (NICU), the early identification of extremely preterm infants who will develop IVH and/or PH is challenging. Clinical signs currently monitored for detection of haemodynamic compromise are blood pressure, heart rate (*HR*) and peripheral capillary oxygen saturation (*SpO*_2_). These clinical signs provide limited information on systemic blood flow and organ perfusion^13^. The recent increase in the use of targeted neonatal echocardiography (TnECHO) provides additional information on cardiac haemodynamics including cardiac outputs and function, direction of shunts (e.g. patent ductus arteriosus (PDA)) and volemic status. However, it does not provide continuous information as it is limited to specific time-point assessments^14^. Accurate and real-time monitoring of blood flow and perfusion would allow to better identify and recognize abnormal circulation in the extremely preterm infant early in life.

In the last decade, several neonatal studies have used conventional near infrared spectroscopy (NIRS) as a non-invasive continuous bedside evaluation of cerebral regional haemoglobin oxygen saturation (*CrSO*_2_)^15^. In extremely preterm infants, associations between low *CrSO*_2_ and IVH^16–18^, ventricular dilation^16,19^ and respiratory distress syndrome have been described^20^. Low *CrSO*_2_ was also associated with brain injury, poor neurodevelopmental outcomes and higher mortality^21^ in this population^22–25^. While *CrSO*_2_ is not a direct measure of blood flow or perfusion, these studies have demonstrated the high potential of NIRS for continuous brain monitoring in extremely preterm infants. Also, pulse oximetry was used to derive peripheral perfusion through the measure of the ratio between pulsatile and non-pulsatile signals^26^. This ratio refers to the peripheral perfusion index (*PI*). This parameter has been described in extremely^27–29^, very^30–32^ or moderate to late^30,33^ preterm infants and healthy term newborns^34,35^. While this technique showed signals variability, *PI* has the potential to monitor systemic blood perfusion in the extremely preterm infant.

Both NIRS *CrSO*_2_ and *PI* signals are displayed in real-time on device screen and are useful in the clinic to detect fast fluctuations and very low values. Previous studies have proposed methods to analyze the haemodynamic signals based on the statistical stationarity of the signals^36–38^. However, in normal or pathological conditions, it is thought that human haemodynamic physiology is non-stationary^39,40^. In addition, it was proposed that slow and prolonged periods of impaired blood flow are considered more detrimental than transient fast fluctuations^41^. In contrast to stationary methods, continuous wavelet analysis allows the decomposition and analysis of non-stationary signals at different time-scales^42,43^. This approach can be used to provide common power of two signals in the time-frequency space by deriving parameters such as cross-correlation, phase, gain (transfer function analysis)^25^ and coherence^44^.

In a previous study in extremely preterm infants, we used time-series of *CrSO*_2_ and preductal *PI* to describe linear correlations with low cardiac output states in the first 72 h of life^29^. Here, we aimed to describe time-frequency relationships between NIRS *CrSO*_2_ and preductal *PI*, *SpO*_2_ and *HR* in extremely preterm infants with haemorrhagic complications (IVH and/or PH), and compare them to healthy controls. We hypothesized that our time-frequency approach allowed to detect haemodynamic instability potentially associated with cerebrovascular autoregulation and haemorrhagic complications observed in extremely preterm infants in the first 72 h of life.

## Results

### Demographics, echocardiography, blood gas analysis and vital monitoring

The PH-IVH group (*N*_*H*_ = 8) included 2 patients with concomitant diagnosis of PH and IVH, 3 patients with a diagnosis of PH only, and 3 patients with a diagnosis of IVH only. One patient with an IVH (grade 3 according to Papile classification)^45^ deceased at day 16 of life secondary to an evolving hydrocephalus and severe respiratory distress syndrome. Also, one patient from the healthy control group deceased at day 28 due to multi-organ failure secondary to necrotizing enterocolitis. While death is an adverse event, they were not excluded from the analysis as death occurred long after the observational study period (first 72 hours of life). Otherwise, all infants in the control group had an uncomplicated first week of life.

Table 1 summarizes demographic in PH-IVH patients and healthy controls as well as blood gas and mean arterial blood pressure (*MABP*) data averaged over the 72 h of life. Gender, antenatal steroids, birth weight, GA, length of stay, APGAR score (at 5 and 10 min) were not significantly different between PH-IVH infants and healthy controls. Respiratory distress syndrome was observed in all preterm infants (*N*_*T*_ = 19). All PH-IVH patients were mechanically ventilated at H6 and 7/8 were still on ventilator at H72. Non-invasive ventilation was more frequently used in the healthy group throughout the study period. Blood gas analysis revealed significantly lower mean haemoglobin concentration in the blood (*HGB*) and *MABP* in PH-IVH patients compared to controls. Other parameters including mean *pH*, partial pressure of carbon dioxide (*PaCO*_2_) and blood lactates were not significantly different.

**Table 1:** Demographic, blood gas values and mean arterial blood pressure variables in patients with a pulmonary (PH) and/or cerebral intraventricular (IVH) haemorrhage and healthy controls (results are expressed as number (%), median and (IQR)).

Variable	PH-IVH patients (*N*_*H*_ = 8)	Healthy controls (*N*_*C*_ = 11)	*p*-value**
Male gender [%]	4/8 (50)	5/11 (46)	0.85
Antenatal steroids [%]	2/8 (25)	6/11 (55)	0.20
Birth weight [g]	710 (655, 780)	910 (600, 1020)	0.14
Gestational age [wk]	24.8 (24.3, 25.3)	25.7 (24.9, 27.4)	0.15
Length of stay [days]	115 (110, 148)	97 (77, 119)	0.26
APGAR			
at 5 min	6 (4, 8)	6 (5, 9)	0.22
at 10 min	8 (6, 9)	7 (6, 9)	0.85
Respiratory distress syndrome [%]	8/8 (100)	11/11 (100)	—
Ventilation parameters at H6 [%]			
CMV and HFOV	8/8 (100)	6/11 (55)	**0.03**
NIV or CPAP	0/8 (0)	5/11 (46)	**0.03**
Ventilation parameters at H24 [%]			
CMV and HFOV	7/8 (88)	6/11 (55)	0.13
NIV or CPAP	1/8 (13)	5/11 (46)	0.13
Ventilation parameters at H48 [%]			
CMV and HFOV	7/8 (88)	8/11 (73)	0.44
NIV or CPAP	1/8 (13)	3/11 (27)	0.44
Ventilation parameters at H72 [%]			
CMV and HFOV	7/8 (88)	4/11 (36)	**0.03**
NIV or CPAP	1/8 (13)	6/11 (55)	0.06
Blood gas analysis*			
*pH*	7.24 (7.21, 7.28)	7.28 (7.24, 7.30)	0.15
*PaCO*_2_ [mmHg]	48.03 (46.35, 51.53)	47.46 (42.83, 50.74)	0.61
*HGB* [g/dl]	120.88 (116.10, 123.73)	137.00 (123.38, 142.71)	**0.03**
Lactates	2.69 (2.17, 3.74)	2.16 (1.80 2.72)	0.10
Mean arterial blood pressure*			
*MABP* [mmHg]	30.47 (28.87, 31.67)	34.36 (32.79, 36.21)	**0.02**

Interquartile range (IQR); conventional mechanical ventilation (CMV); high frequency oscillation ventilation (HFOV); non-invasive ventilation (NIV); Continuous positive airway pressure (CPAP); haemoglobin concentration in the blood (*HGB*); partial pressure of carbon dioxide (*PaCO*_2_); mean arterial blood pressure (*MABP*). *Median values of blood gas analyses and *MABP* measurements averaged over the 72 h of life. **p-values are generated with statistical comparisons of the means using general linear mixed models adjusted with Bonferroni correction.

Echocardiographic measurements and PDA parameters are shown in Table 2. PDA diameter (at H6, H24 and H48) and PDA treatment were not significantly different between PH-IVH infants and controls while PDA diameter at H72 and PDA closure were significantly lower and higher, respectively, in controls. Echocardiographic measurements showed significantly lower left ventricular output (LVO) and right ventricular output (RVO) at H6 in PH-IVH infants compared to controls. None of the comparison with superior vena cava (SVC) flow, left ventricular ejection fraction (LFEF) and left ventricular shortening fraction (LVSF) was significantly different between the two groups.

**Table 2:** Echocardiographic variables in patients with a pulmonary (PH) and/or cerebral intraventricular (IVH) haemorrhage and healthy controls (results are expressed as number (%), median and (IQR)).

Variable	PH-IVH patients (*N*_*H*_ = 8)	Healthy controls (*N*_*C*_ = 11)	*p*-value**
PDA diameter [mm]			
at H6	1.55 (1.40, 1.95)	1.58 (1.30, 1.90)	0.76
at H24	1.80 (1.49, 1.90)	1.40 (1.29, 1.80)	0.20
at H48	1.60 (1.46, 2.00)	1.5 (1.15, 1.80)	0.19
at H72	1.53 (1.50, 2.00)	1.30 (0.00 1.60)	**0.04**
PDA treatment <72 h of life [%]	3/8 (38)	3/11 (27)	0.64
PDA closure	0/8 (0)	5/11 (46)	**0.03**
TnECHO Measurements at H6			
LVO [mL/kg/min]	49.83 (36.91, 70.28)	136.00 (68.00, 208.73)	**0.01**
RVO [mL/kg/min]	80.58 (49.74, 178.67)	195.94 (186.45, 296.00)	**0.02**
SVC flow [mL/kg/min]	43.26 (13.81, 68.19)	46.97 (30.79, 93.00)	0.26
LVEF [%]	53.65 (45.45, 64.20)	58.70 (52.50, 67.40)	0.40
LVSF [%]	24.55 (19.90, 31.00)	28 (23.90, 33.30)	0.36
TnECHO Measurements at H24			
LVO [mL/kg/min]	113.54 (61.08, 197.00)	154.00 (100.74, 246.00)	0.31
RVO [mL/kg/min]	188.80 (79.55, 304.45)	310.00 (211.00, 437.00)	0.13
SVC flow [mL/kg/min]	42.88 (33.05, 94.00)	66.00 (52.00, 101.23)	0.24
LVEF [%]	68.90 (62.50, 73.40)	66.60 (61.60, 69.90)	0.65
LVSF [%]	34.20 (30.10, 38.00)	32.50 (29.50, 35.50)	0.72
TnECHO Measurements at H48			
LVO [mL/kg/min]	141.27 (74.79, 172.06)	179.45 (134.00, 243.00)	0.13
RVO [mL/kg/min]	224.71 (104.77, 376.34)	280.82 (152.30, 354.00)	0.39
SVC flow [mL/kg/min]	50.82 (30.03, 86.76)	84.52 (65.00, 90.00)	0.41
LVEF [%]	70.50 (64.40, 75.40)	70.50 (67.00, 73.00)	0.85
LVSF [%]	36.00 (31.50, 39.70)	35.60 (33.30, 38.90)	0.98
TnECHO Measurements at H72			
LVO [mL/kg/min]	139.44 (102.48, 182.00)	192.00 (177.00, 225.14)	0.11
RVO [mL/kg/min]	218.00 (162.79, 352.00)	323.63 (169.97, 407.00)	0.41
SVC flow [mL/kg/min]	56.00 (37.53, 72.39)	76.90 (23.77, 123.00)	0.27
LVEF [%]	75.40 (72.40, 81.40)	69.90 (66.50, 74.50)	0.14
LVSF [%]	40.00 (37.70, 45.80)	35.60 (34.10, 39.40)	0.16

Interquartile range (IQR); patent ductus arteriosus (PDA); targeted neonatal echocardiography (TnECHO); left ventricular output (LVO); right ventricular output (RVO); superior vena cava (SVC); left ventricular ejection fraction (LVEF); left ventricular shortening fraction (LVSF). **p-values are generated with statistical comparisons of the means using general linear mixed models adjusted with Bonferroni correction.

### Cerebral NIRS and peripheral oximetry in PH-IVH patients versus healthy controls

**Figure 1:**
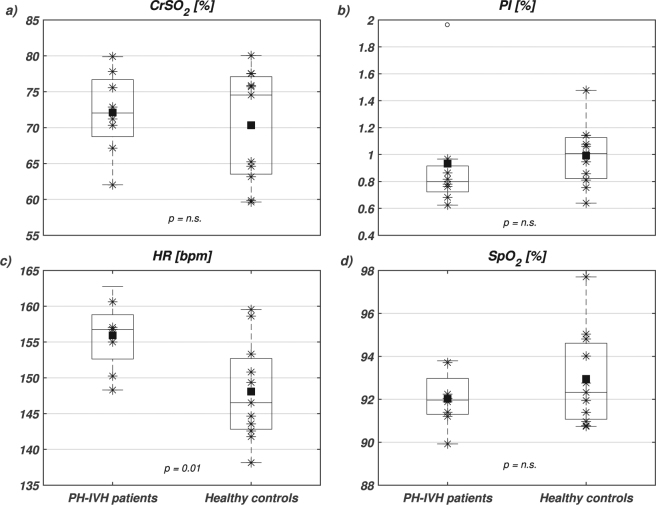
Boxplots of (**a**) near infrared spectroscopy (NIRS) cerebral regional haemoglobin oxygen saturation (*CrSO*_2_), (**b**) peripheral perfusion index (*PI*), (**c**) heart rate (*HR*) and (**d**) capillary oxygen saturation (*SpO*_2_) in PH-IVH patients (left boxplots) and healthy age-matched controls (right boxplots). On each box, the central mark is the median, the square is the mean, the stars are the individual data, the edges of the box are the 25^th^ and 75^th^ percentiles, and the whiskers show the 95% confidence interval. Empty circles denote outliers and statistical comparisons are indicated with corresponding *p*-values (n.s., non-significant).

Figure 1 displays boxplot distributions of averaged *CrSO*_2_, *PI*, *HR* and *SpO*_2_ in PH-IVH patients and healthy controls. None of the parameters was significantly different except *HR*, which was significantly higher in PH-IVH patients compared to controls.

**Figure 2:**
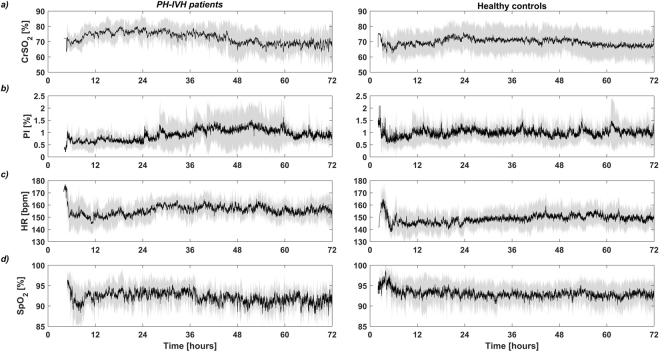
Temporal distributions of (**a**) near infrared spectroscopy (NIRS) cerebral regional haemoglobin oxygen saturation (*CrSO*_2_), (**b**) peripheral perfusion index (*PI*), (**c**) heart rate (*HR*) and (**d**) capillary oxygen saturation (*SpO*_2_) in PH-IVH patients (left column) and healthy age-matched controls (right column) in the first 72 h of life. On each plot, the black curve is the mean and the grey shaded region represents one standard deviation of the group.

The temporal distributions of *CrSO*_2_, *PI*, *HR* and *SpO*_2_ in PH-IVH patients and healthy controls are shown in Fig. 2. The black line shows the average between subjects while the grey shaded region shows one standard deviation over the 72 h of monitoring. In PH-IVH patients, *CrSO*_2_ increased in the first 24 h of age while slowly decreasing until 48 h and reaching a plateau from 48 to 72 h of age. This trend was slightly different in healthy controls where *CrSO*_2_ was around 70% for the total monitoring period. In contrast to *CrSO*_2_, *PI* showed a slow increase between 24 and 48 h in PH-IVH infants while remained mostly constant in healthy controls over the first 72 h. As displayed in Fig. 1, mean *HR* was significantly higher in PH-IVH infants compared to controls and this result can be qualitatively observed in the temporal distribution. Over the total monitoring period, *SpO*_2_ showed slightly higher values in healthy controls compared to PH-IVH infants while mean *SpO*_2_ was not significantly different as shown in Fig. 1(d).

**Figure 3:**
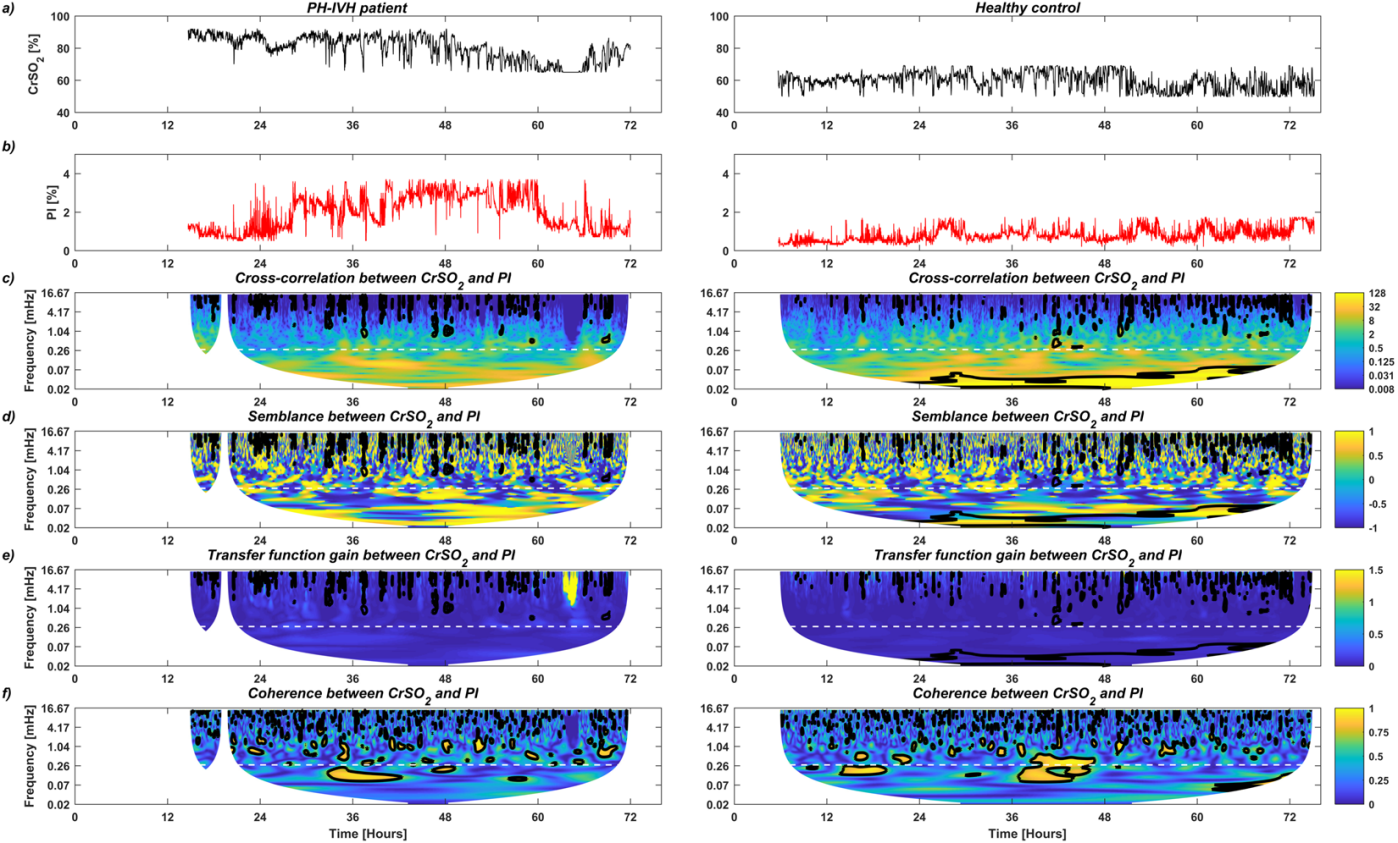
Example of the complete analytical workflow in a healthy control (left column) and in an infant with pulmonary (PH) and/or intraventricular haemorrhage (IVH, right column): (**a**) and (**b**) depict temporal distributions of near infrared spectroscopy (NIRS) cerebral regional haemoglobin oxygen saturation (*CrSO*_2_) and peripheral perfusion index (*PI*) in the first 72 h of life, respectively; (**c**–**f**) display the amplitude of the cross-correlation, the semblance (anti-phase and in-phase), the amplitude of the gain (transfer function) and the coherence between *CrSO*_2_ and *PI* in the time-frequency space. Regions that are statistically significant are comprised in a black bold contour. A dashed white line indicates the selected ultra-frequency band of slow and prolonged periods of >1 h (<0.28 mHz) used for statistical analysis. Regions outside the cone of influence in which data are not used in the statistical analysis below are not shown.

Figure 3 illustrates a graphical example of the complete analytic workflow in a PH-IVH patient (left column) and a healthy control (right column). Figure 3(a,b) show the temporal distributions of *CrSO*_2_ and *PI* in the first 72 h, respectively. Figure 3(c–f) show the amplitude of the cross-correlation, the semblance, the amplitude of the gain and the coherence between *CrSO*_2_ and *PI* in the time-frequency space (frequency range <0.28 mHz, equivalent to slow and prolonged periods of >1 h, as indicated by the dashed white line), respectively. Figure 3(c–f) display regions that are statistically significant (comprised in a black bold contour). Regions outside the cone of influence in which data are not used in the statistical analysis below are not shown.

Table 3 describes the percentage of time of significant cross-correlation, semblance (anti-phase and in-phase), gain and coherence calculated for *CrSO*_2_ with *PI*, *SpO*_2_ and *HR* in patients with PH-IVH versus healthy controls. Percentages of significant cross-correlation between *CrSO*_2_ and peripheral oximetry (*PI*, *SpO*_2_ and *HR*) were all significantly lower in PH-IVH infants compared to controls. Only the percentages of anti-phase and in-phase semblance between *CrSO*_2_ and *HR* were significantly higher and lower, respectively, in PH-IVH infants compared to controls. Also, the percentage of significant gain between *CrSO*_2_ and *HR* was significantly lower in PH-IVH infants than in controls. Results for coherence were not significantly different between the two groups.

**Table 3:** Wavelet decomposition parameters (amplitude of the cross-correlation, semblance, gain and coherence) calculated between near infrared spectroscopy (NIRS) cerebral regional haemoglobin oxygen saturation (*CrSO*_2_) and peripheral oximetry parameters, including perfusion index (*PI*), capillary oxygen saturation (*SpO*_2_) and heart rate (*HR*).

Variable	PH-IVH patients (*N*_*H*_ = 8)	Healthy controls (*N*_*C*_ = 11)	*p*-value**
Cross-correlation (*W*_*xy*_) [%]			
between *CrSO*_2_ and *PI*	11.99 (4.75, 25.05)	32.33 (29.84, 36.01)	**0.002**
between *CrSO*_2_ and *SpO*_2_	10.44 (3.25, 14.17)	26.81 (20.96, 32.27)	<**0.001**
between *CrSO*_2_ and *HR*	6.06 (2.23, 16.62)	25.95 (22.86, 33.27)	**0.006**
Anti-phase semblance (${S}_{xy}{|}_{{\rm{\Delta }}{\varphi }_{xy}=\pi \pm \pi \mathrm{/4}}$) [%]			
between *CrSO*_2_ and *PI*	23.24 (15.81, 29.24)	25.86 (21.98, 33.50)	0.246
between *CrSO*_2_ and *SpO*_2_	24.34 (19.31, 26.58)	26.57 (21.41, 34.41)	0.126
between *CrSO*_2_ and *HR*	16.32 (13.49, 21.65)	10.55 (7.68, 11.65)	**0.047**
In-phase semblance ${S}_{xy}{|}_{\Delta {\varphi }_{xy}=\pm \pi \mathrm{/4}}$) [%]			
between *CrSO*_2_ and *PI*	27.91 (21.84, 32.01)	19.35 (16.38, 23.56)	0.076
between *CrSO*_2_ and *SpO*_2_	22.53 (19.88, 29.10)	23.45 (17.27, 26.82)	0.577
between *CrSO*_2_ and *HR*	38.67 (35.93, 44.09)	47.36 (43.63, 54.48)	**0.040**
Gain (*H*_*xy*_)			
between *CrSO*_2_ and *PI*	0.04 (0.02, 0.04)	0.06 (0.04, 0.08)	0.062
between *CrSO*_2_ and *SpO*_2_	0.31 (0.27, 0.50)	0.42 (0.33, 0.45)	0.616
between *CrSO*_2_ and *HR*	0.78 (0.58, 1.00)	1.26 (0.80, 1.41)	**0.045**
Coherence $({R}_{xy}^{2})$ [%]			
between *CrSO*_2_ and *PI*	4.96 (4.12, 12.07)	5.95 (2.47, 11.97)	0.926
between *CrSO*_2_ and *SpO*_2_	11.96 (9.23, 17.52)	10.76 (6.44, 17.03)	0.621
between *CrSO*_2_ and *HR*	11.26 (9.25, 18.22)	19.86 (16.42, 23.00)	0.074

For each pair of signals, the percentage of time of significant cross-correlation (*W*_*xy*_), semblance (*S*_*xy*_), gain (*H*_*xy*_) and coherence $({R}_{xy}^{2})$ between any two signals were summed over the 72 h period (for frequencies <0.28 mHz). Comparisons are provided between patients with a pulmonary (PH) and/or cerebral intraventricular (IVH) haemorrhage, and healthy controls (median and (IQR)).

Interquartile range (IQR).

**p-values are generated with statistical comparisons of the means using general linear mixed models adjusted with Bonferroni correction.

Supplementary Table S1 online includes the entire list of *p*-values generated when reproducing the previous analysis with GA, birth weight, length of stay, *pH*, *PaCO*_2_, *HGB* or lactates as an individual independent covariate. The percentages of significant cross-correlation between *CrSO*_2_ and peripheral oximetry (*PI*, *SpO*_2_ and *HR*) remained significantly lower in PH-IVH infants compared to controls. However, the percentages of anti-phase semblance, in-phase semblance and gain between *CrSO*_2_ and *HR* were not systematically kept significantly different between PH-IVH infants and controls.

## Discussion

In this prospective observational study of 19 infants born <28 wk gestational age, we showed that cerebral NIRS and peripheral oximetry parameters acquired in the first 72 h of life share time-frequency relationships in infants with PH and/or IVH that differ from healthy controls. These relationships were described using wavelet decomposition that allowed deriving common time-frequency information between simultaneous recordings of *CrSO*_2_, preductal *PI*, *SpO*_2_ and *HR*. To our knowledge, this is the first study in extremely premature infants reporting time-frequency analysis of simultaneous measurements of NIRS and preductal *PI* performed in the first 72 h of life. These differences may reflect haemodynamic instability associated with cerebrovascular autoregulation and the vulnerability to PH and/or IVH observed during the transitioning physiology.

The lower percentage of significant cross-correlation between *CrSO*_2_ and *PI* observed in PH-IVH patients compared to healthy controls may reflect their more prominent haemodynamic instability. After birth in the extremely preterm infant, there is a temporary left ventricular dysfunction due to changes in loading conditions faced by the immature myocardium^3,4^. This temporary left ventricular dysfunction was likely represented by significantly lower LVO and RVO and by lower LVEF and LVSF (not significant) at H6 in the PH-IVH group. The increase in left ventricular afterload combined with the left ventricular dysfunction result in increased left atrium pressure and pulmonary venous pressure in the pulmonary veins. These increases will then augment the pressure and the flow in the pulmonary vasculature. The risk of PH in the lungs is increased when these fluctuations are accompanied by factors including the immaturity of the lungs, surfactant deficit and fast decreases in ventilation pressures. The risk of PH becomes even higher when considering the increase of the pulmonary blood flow through the left-to-right shunt via a haemodynamically significant PDA^46^. Significant PDA in the extremely preterm infant was associated with PH^11^ and IVH^47^, as well as mortality^48^. The measure of the PDA diameter is thought to be a marker of PDA severity^49^ and treatment with nonsteroidal anti-inflammatory drugs (indomethacin or ibuprofen) reduced the PDA size in neonates with birth weight <1 kg^50^. In addition, indomethacin was shown to reduced cerebral blood flow (*CBF*) while maintaining cerebral oxygen metabolism through oxygen extracted fraction compensation^51^. In our groups, 3 infants of each group received ibuprofen treatment during their first 72 h of life, as ibuprofen is less prone to influence *CBF* than indomethacin^52^. Interestingly at H6, PH-IVH patients and healthy controls had similar median PDA diameters while at H24, H48 and H72, PH-IVH infants showed higher median PDA diameters compared to healthy controls. While PDA difference was only significant at H72, these observations are concordant with the potential role of significant PDA on IVH and PH in the extremely preterm infant.

The high incidence of IVH occurring in the first 3 days of life is also related to the increase in left ventricular afterload and secondary left ventricular dysfunction^47^. The initial low cardiac outputs lead to periods of ischemia followed by reperfusion with improvement of the ventricular function with time^5^. These ischemia-reperfusion events will in turn increase the risk of IVH due to the immature and thin cerebral vasculature that has limited ability to respond to abrupt fluctuations^23^. In controls, the gradual increase in LVO through the first 72 h of life was consistent with a prior study^53^, while this gradual increase stopped at H48 in PH-IVH infants. This observation is concordant with *CrSO*_2_ plateauing at 48 h as well as higher variability found in *PI*. In addition, head ultrasound was only performed once in the first 72 h of life, which did not allow to reveal the exact time of occurrence and thus its categorization in early or late haemorrhage was not possible^23^. While these observations appear to be related in time, the current data does not allow to speculate on potential physiological mechanisms.

In extremely preterm infants, it is recommended to maintain *SpO*_2_ within the range of 88–92%. This range is associated to a minimized risk of mortality as it acts to decrease the concentration of oxygen free radicals^54^. The goal to maintain this range in sicker infants is more challenging to reach and may lead to additional and higher physiological fluctuations. This practice combined with the unstable status in sicker infants may be associated to the lower percentage of significant cross-correlation between *CrSO*_2_ and *SpO*_2_ observed in PH-IVH patients compared to healthy controls. Therefore, longer periods of significant cross-correlation between *CrSO*_2_ and *SpO*_2_ in the healthy infants may reflect a better adaptation and higher tolerance to transition from fetal to postnatal circulation.

In this study, we have focused on monitoring NIRS *CrSO*_2_ as a validated estimate of *CBF*^55–57^ and peripheral perfusion index as a surrogate marker for circulatory status^30^. We speculate that longer periods of significant cross-correlation between *CrSO*_2_ and *PI* represent a lower risk of haemorrhagic complications and altered autoregulation in the first 72 h of life in extremely preterm infants. We support this hypothesis with complementary findings, i.e. higher cross-correlation in *CrSO*_2_ with *SpO*_2_ and *HR* as well as higher in-phase semblance and gain between *CrSO*_2_ and *HR* in healthy controls compared to PH-IVH infants. In the same vein, shorter time periods of significant anti-phase semblance were observed in healthy controls compared to PH-IVH infants, which reinforces our postulate.

In previous studies, time-frequency parameters (cross-correlation, semblance, gain and coherence) were used to characterize autoregulation and predict clinical outcome. Our results are consistent with studies that reported an association between higher coherence or gain between *CrSO*_2_ and *MABP*, healthy status and good outcome^41,58,59^. However, high coherence or gain between *CrSO*_2_ and *MABP* (or *HR*) were also associated with increased incidence of IVH, brain injury and altered autoregulation^23,25,37,41,59–62^. The comparison of our findings with these studies is however limited by the fact they were based on the relationship between *CrSO*_2_ and *MABP* (or *HR*)^23,25,58–61,63,64^. Indeed, our study included recordings of only one measure of *MABP* per hour, which was not sufficient to decompose accurately signals in the time-frequency space. Our data only showed that PH-IVH patients had a significantly lower average *MABP* than in controls over the observational period.

In addition, this inconsistency throughout literature is certainly multifactorial and related to the selection of specific methodological approaches to measure autoregulation^65^ (see discussion below) as well as the fundamental definition of cerebrovascular autoregulation, as recently discussed by Stammwitz *et al*.^58^. Originally, autoregulation was defined as a neuroprotective mechanism leading to changes in the muscular wall of blood vessels in response to *MABP* fluctuations^65^. Intact autoregulation limits *CBF* changes over a range of cerebral perfusion pressures, which allows appropriate delivery of oxygen to the brain. In the extremely preterm infants, this mechanism is thought to be limited or disrupted^41^. Novel concepts are emerging^64^ and one of these proposed to define autoregulation as a response to metabolic demand^66^. However, as highlighted by Stammwitz *et al*.^58^, this concept also assumes an association between higher coherence and altered autoregulation, which is inconsistent with our findings. In addition, our experimental protocol did not allow to assess the cerebral metabolic demand (see below for a potential approach).

The selection of the frequency band is of great importance to assess cerebrovascular autoregulation (see this recent review)^67^ and may partially explain the inconsistency in literature. Here, the frequency band (<0.28 mHz) was selected to capture slow and prolonged periods of *CBF* fluctuations that are considered more detrimental than transient and fast fluctuations^41,59,67^. This approach was applied in neonatal hypoxic ischemic encephalopathy^43^ and extremely preterm infants^41,58^. In other studies, ultra-low (3–20 mHz) and very-low (20–50 mHz) frequency ranges as well as Mayer wave (80–120 mHz) ranges were used to measure autoregulation^25,59,63,64,68^. To compare with literature, we reproduced our analysis within the ultra-low frequency band (between 0.28 and 20 mHz). However, none of the relationships between *CrSO*_2_ and *HR* was significant while only cross-correlation between *CrSO*_2_ and *PI* remained significantly lower in PH-IVH infants compared to controls. This additional analysis suggests that cross-correlation between *CrSO*_2_ and *PI* may better reflect haemorrhagic complications associated with slow and transient haemodynamic fluctuations, compared to relationships with *HR*.

Due to the low number of patients in our study, our findings were likely to be influenced by confounders. Indeed, our statistical analysis failed to provide systematically significant difference in semblance and gain when adjusting for GA, birth weight, length of stay, *pH*, *PaCO*_2_, *HGB* or lactates as individual independent covariate. However, cross-correlation between *CrSO*_2_ and *PI* (as well as with *SpO*_2_ and *HR*) remained systematically significantly lower in PH-IVH infants compared to controls. This analysis suggests that cross-correlation may be the most robust marker (as compared to gain^68^, semblance or coherence) of haemodynamic instability and vulnerability to haemorrhages observed during the transitioning physiology.

Monitoring of *CrSO*_2_ has great potential for continuous long-period monitoring in the extremely preterm infant. However, *CrSO*_2_ remains an estimate of *CBF* and when used alone may not differentiate particular pathological conditions to healthy status. Recent advances in bedside monitoring techniques such as frequency-domain NIRS (FDNIRS) and diffuse correlation spectroscopy (DCS) have shown the ability to monitor an index of *CBF* (*CBF*_*i*_) in addition to absolute haemoglobin oxygen saturation (*SO*_2_), which can be used to derive an index of the cerebral metabolic rate of oxygen consumption (*CMRO*_2*i*_). This technique was used to monitor premature infants in the first weeks of life^69–73^, and more recently to show lower *CMRO*_2*i*_ and *CBF*_*i*_ in preterm infants with low-grade germinal matrix-IVH^74^. Furthermore, advanced NIRS methods were useful to monitor hypothermia therapy in neonatal hypoxic-ischemic encephalopathy^75–77^ and perioperative haemodynamics in neonatal congenital heart disease undergoing surgery^78–83^. These techniques showed great potential to monitor the effect of an intervention and will be useful in decision making for haemodynamic compromise treatment in extremely preterm infants. This approach may also be relevant to investigate the new concept of autoregulation defined as a response to metabolic demand.

Limitations include the restriction in studying PH and IVH individually. However as exposed above, the cardiac dysfunction that is associated with the transitioning physiology is thought to be, among other factors, partially responsible for the development of both PH and IVH. Mechanistically and physiologically, there is an interest to study haemorrhagic complications in the extremely preterm infants. Another limitation is that cerebral NIRS measurements were performed in the frontal lobe and may not be representative of whole brain physiology. While Wijbenga *et al*. observed no substantial difference in *CrSO*_2_ between brain regions^84^, other studies demonstrated haemodynamic regional asymmetries^71^. In addition, a study reported that NIRS sensor for neonates provided higher values than the adult one^85^. However, as the amplitude of *CrSO*_2_ was not a comparison of interest between the two groups, this limitation had limited effect. The lack of significant difference in *PaCO*_2_ between the two groups may be due to the sampling rate of recordings (every 6–8 h). While a recent study showed that acute *PaCO*_2_ fluctuations affect cerebral oxygenation^86^, our methodology did not allow to detect those *PaCO*_2_ fluctuations. The measure of the PDA diameter with TnECHO may also lack of accuracy as the diameter is likely to dynamically change slightly from a time to another. However, the trends reported in our study are consistent with PDA diameters reported in the literature^49^. Also, due to edge artifacts inherent to the wavelet decomposition, partial information (at very-low frequencies) associated to the beginning and the end of the experimental period were lost. This limitation is also related to the minimal duration of monitoring required to perform the analysis. As slow and prolonged periods of haemodynamic instability (<0.28 mHz) were of interest, reducing the total duration of monitoring has a direct impact on the resulting frequency band of interest. In particular, when reducing the monitoring period to 48, 24, and 12 h, the corresponding lowest available frequency increased to 0.017, 0.033, and 0.07 mHz, respectively. As an example, our analysis was reproduced for data monitored between 24 and 48 h of life and only cross-correlation and in-phase semblance between *CrSO*_2_ and *HR* remained significant. This additional analysis shows the limitation of our approach in investigating slow and prolonged periods of haemodynamic instability and the determination of the precise minimal monitoring duration has to be investigated.

In summary, as opposed to previous studies, we focused on simultaneous monitoring of NIRS *CrSO*_2_ as an estimate of *CBF* and preductal *PI* as a surrogate for circulatory status. This technique was used to compare extremely premature infants with PH and/or IVH to healthy controls in their first 3 days of life. We decomposed the monitoring signals in the time-frequency plane allowing to assess their common power for slow and prolonged periods of time. This method appeared to be particularly relevant in extremely preterm infants who develop PH and/or IVH as significant differences were observed compared to healthy age-matched controls. These differences may reflect haemodynamic instability associated with cerebrovascular autoregulation and haemorrhages observed during the transitioning physiology. Our results also suggest that cross-correlation was the most robust parameter to differentiate PH-IVH infants to healthy controls. This study may help to better understand physiological changes during the transitioning period and this continuous assessment has high potential to further monitor response to treatment in these patients.

## Methods

### Patients

Twenty premature infants born <28 weeks of gestation were enrolled in a prospective observational study at the tertiary NICU of the Sainte-Justine Hospital University Centre (affiliated to University of Montréal, Montréal, QC, Canada) between July 2015 and May 2016. The study was approved with institutional review board from the *Comité d'éthique de la recherche* at CHU Sainte-Justine, named by the Québec Government (#FWA00021692) and acts in accordance with Québec and Canada laws, and the Code of Federal Regulations in the USA. Also, methods used in this study were carried out in accordance with the previous guidelines and regulations. Parental written informed consent was obtained in the 24 h prior birth or within the first 4–6 hours of life. Infants with congenital heart defects (excluding PDA and septal defects), multiple congenital anomalies and/or moribund clinical state were excluded. One recruited preterm infant was further excluded from the analysis due to inability to obtain prolonged periods of good quality data. Thus, 19 preterm infants (*N*_*T*_ = 19) were included for data analysis.

Patients were subdivided in two groups: 1) extremely preterm infants (*N*_*H*_ = 8) with a diagnosis of PH (as defined by the presence of blood in the trachea and increase needs of oxygen requirement and/or ventilation parameters)^46^ and/or IVH of grade 2 and higher^45^ (together defined as the PH-IVH group) in the first 72 hours of life, and 2) healthy controls (*N*_*C*_ = 11) with no PH and no IVH in the first 72 hours of life.

Three infants of each group received nonsteroidal anti-inflammatory drugs (NSAIDs, here ibuprofen) to treat PDA. Three healthy controls who received NSAIDs were successful in the closure of the PDA while two healthy controls had closure of the PDA spontaneously. None of the patient was sedated during the study period.

### Cerebral NIRS and peripheral oximetry monitoring

Patients were monitored with NIRS measure of *CrSO*_2_ (INVOS 5100, Covidien, MI, USA) and peripheral oximetry (Radical 7 Pulse, Masimo, Irvine, CA, USA) measures of *PI*, *SpO*_2_ and *HR*. Multimodal monitoring was applied until 72 h (standard deviation 2 h) of life. NIRS *CrSO*_2_ was monitored in the right frontal location at a sampling rate of 2 recordings per minute while peripheral oximetry was positioned on the right hand (preductal) and recorded every 5 seconds.

Antenatal, birth and neonatal characteristics were collected in addition to neonatal outcomes from the medical charts. *HGB* (g/dl), lactates, *pH* and *PaCO*_2_ (mmHg) were derived from blood gas analysis performed every 6–8 h in the first 72 h of life according to our institutional protocol for extremely preterm infants. Blood pressure was monitored using an indwelling arterial catheter when present or using a pressure transducer. However, *MABP* was only recorded every hour (or more frequently if needed). Echocardiographic measurements of LVO, RVO, LFEF, LVSF, SVC flow, PDA characteristics and ventilation parameters were collected at 6, 24, 48 and 72 hours of life (noted here by H6, H24, H48 and H72, respectively). The method used for those echocardiographic measurements was previously reported^29^.

### Data preprocessing

Prior to data analysis, *CrSO*_2_, *PI*, *SpO*_2_ and *HR* were manually inspected to identify artefacts that were defined as signal variation greater than 1.5 standard deviation from the averaged signal. Artefacts that were described by a high amplitude transient change were attenuated by linear interpolation. Artefacts that occurred for a duration of more than 5 minutes were removed from the original signals. As NIRS and oximetry artefacts occurred at different periods, the removed temporal segments from one signal were mirrored in the other signal when calculating common power. All signals were further downsampled to 0.033 Hz to match the lowest temporal resolution, which corresponded to NIRS data.

### Data analysis with wavelet decomposition

Wavelet decomposition allowed to describe the temporal signal in the time-frequency space. It allowed the analysis of localized variations of power in the signal. To decompose the signal in a range of frequencies, a continuous wavelet transform (CWT) was applied to the temporal signal by convolving it with a stretched and translated function *ψ*_0_, called the complex mother wavelet^42^. The *Morlet* mother wavelet (Ω_0_ = 6) was used for the wavelet analysis as it provided a good balance of time-frequency localization^44^ and was previously used with NIRS data^87^. Therefore, the CWT of a time-series *x*(*n*) of length *N* with a uniform temporal sampling rate *δt* was defined by

1 $${W}_{x}(n,s)=\sqrt{\frac{\delta t}{s}}\sum _{n^{\prime} =1}^{N}{x}_{n}{\psi }_{0}((n^{\prime} -n)\frac{\delta t}{s})$$

where *n* and *s* were the index of time and scale, respectively. The mother wavelet was not completely localized in time and its application through the CWT produced edge artefacts. To avoid these artefacts, a cone of influence was used to delineate regions of the wavelet spectrum where edge effects are important. The power spectrum beyond this region ensured that edge discontinuities were negligible^42^.

As for conventional Fourier analysis, the wavelet cross-correlation was used to quantify the common power and the relative phase between two signals. The wavelet cross-correlation *W*_*xy*_(*n*,*s*) for two signals *x*(*n*) and *y*(*n*) was defined by

2 $${W}_{xy}(n,s)={W}_{x}{W}_{y}^{\ast }$$

where the operator * denoted the complex conjugate^42,88^. The amplitude of the common power was then given by the absolute value of the wavelet cross-correlation (|*W*_*xy*_|) and its relative phase (Δ*ϕ*_*xy*_) by its argument, such that

3 $${\rm{\Delta }}{\varphi }_{xy}={\tan }^{-1}(\frac{{\rm{\Im }}({W}_{xy})}{{\rm{\Re }}({W}_{xy})})$$

To visualize the phase between −1 and 1, we applied the cosinus function to Δ*ϕ*_*xy*_ and obtained the semblance *S*_*xy*_(*n*,*s*), such that

4 $${S}_{xy}(n,s)=\,\cos ({\rm{\Delta }}{\varphi }_{xy})$$

The semblance tended to 1 when two time-series were correlated in phase and to −1 when they were inversely correlated (anti-phase)^39^. Values were then separated in anti-phase semblance $({S}_{xy}{|}_{{\rm{\Delta }}{\varphi }_{xy}=\pi \pm \pi /4})$ and in-phase semblance $({S}_{xy}{|}_{{\rm{\Delta }}{\varphi }_{xy}=\pm \pi /4})$.

The linear relationship between variations of two time-series was evaluated using the wavelet transfer function, defined as

5 $${H}_{xy}(n,s)=\frac{F(\frac{1}{s}{W}_{xy}(n,s))}{F(\frac{1}{s}{W}_{xx}(n,s))}$$

where *F* was a smoothing operator that allowed to remove singularities by using a moving-window weighted average in time convolved with a boxcar function in scale^42,43,89^. The term *W*_*xx*_ was defined by the wavelet autocorrelation function of *x*(*n*)^43,44^. The amplitude of the transfer function (|*H*_*xy*_|) represented the gain between the time-series. The wavelet decomposition was also useful to quantify the coherence between two signals, which measured the cross-correlation as a function of frequencies. The coherence ${R}_{xy}^{2}(n,s)$ was defined by the square of the amplitude of the common power normalized by the individual power spectra such that

6 $${R}_{xy}^{2}(n,s)=\frac{{|F(\frac{1}{s}{W}_{xy}(n,s))|}^{2}}{F(\frac{1}{s}{|{W}_{x}(n,s)|}^{2})\cdot F(\frac{1}{s}{|{W}_{y}(n,s)|}^{2})}$$

#### Quantification of wavelet parameters

Wavelet decomposition is offering a theoretical framework to quantify the wavelet parameters with a statistical level of significance. For cross-correlation, the level of significance was derived by assuming that the wavelet spectra were *χ*^2^-distributed. Following the analytical formulation previously reported^42^, the distribution of the cross-correlation between *x*(*n*) and *y*(*n*) was significant when

7 $$\frac{|{W}_{x}(n,s){W}_{y}^{\ast }(n,s)|}{{\sigma }_{x}{\sigma }_{y}}\ge \frac{{Z}_{\nu }(p)}{\nu }\sqrt{{P}_{x}{P}_{y}}$$

where *σ*_*x*_ and *σ*_*y*_ were the signal standard deviations and *P*_*x*_ and *P*_*y*_ the respective theoretical power spectra of *x*(*n*) and *y*(*n*), respectively. Here, *Z*_*v*_(*p*) was the confidence interval for a probability *p*. The degrees of freedom *v* of the *χ*^2^-distributions were equal to 2 as we decomposed the signals in complex wavelets. For 95% confidence interval, *Z*_2_(0.95) = 3.999.

There is currently no analytical formulation to derive the statistical significance level for the phase and the amplitude of the transfer function (gain). However, as these quantities were derived from the calculation of the cross-correlation, the level of significance for cross-correlation was used for further statistical comparisons. Other groups also employed this technique^43,44^.

The calculation of the level of statistical significance for the coherence required a specific methodology based on Monte Carlo simulations of a stochastic random Gaussian process. This approach allowed comparing the measured signal to random noise. To model this random noise, a large set of simulated data pairs was generated using a first-order autoregressive model^44^. For the statistical analysis described below, 1000 pairs of data were simulated for each monitoring signal in each patient. Then, the coherence was computed for each simulated pair of signals and the wavelet coherence significance level was estimated by comparing the coherence of the measured signals to the coherence of the simulated data^90^. These computations were performed using an available Matlab (Mathworks, Natick, MA, USA) toolkit^44^. Prior to use the toolkit, we verified that each temporal signal was normally distributed.

### Statistical analysis

In a first analysis, demographics (gender, gestational age, antenatal steroids, birth weight, APGAR scores), echocardiographic measurements, blood gas values, *MABP*, and ventilation parameters in PH-IVH patients were compared to healthy controls using general linear mixed models. Comparisons of variables defined by binary values were performed with a *χ*^2^-test.

In a second analysis, NIRS *CrSO*_2_ and preductal *PI*, *SpO*_2_ and *HR* in PH-IVH patients were compared to healthy controls using general linear mixed models. Then, parameters derived from the wavelet decomposition (cross-correlation, semblance, gain and coherence) were calculated between *CrSO*_2_, *PI*, *SpO*_2_ and *HR*. For each pair of signals, the percentage of time of significant cross-correlation, semblance, gain and coherence between the two signals was averaged for ultra-low frequencies (<0.28 mHz). Then, the percentages were averaged in both groups. Wavelet parameters were then compared between groups using general linear mixed models. These analyses were reproduced when considering GA, birth weight, length of stay, *pH*, *PaCO*_2_, *HGB* and lactates as individual independent covariate. For all group comparisons, a level of significance of 0.05 was used and *p*-values were adjusted with Bonferroni correction where appropriate.

### Data availability

The datasets generated and/or analyzed during the current study are available from the corresponding author on reasonable request.

## Electronic supplementary material

Supplementary Table S1
