# Supplementary material for: Cerebral oxygen saturation and peripheral perfusion in the extremely premature infant with intraventricular and/or pulmonary haemorrhage early in life
Source: Sci Rep. 2018 Apr 25;8:6511. doi: 10.1038/s41598-018-24836-8 (PMC5916916; doi:10.1038/s41598-018-24836-8)
Supplement: Supplementary file 1 — Supplementary Table S1 [file 41598_2018_24836_MOESM1_ESM.pdf]

Cerebral oxygen saturation and peripheral perfusion in the extremely premature infant with intraventricular and/or pulmonary haemorrhage early in life

Thierry P. Beausoleil[1,2], Marie Janaillac[3], Keith J. Barrington[2,3], Anie Lapointe[3,+], and Mathieu Dehaes[2,4,+,*]

[1]Institute of Biomedical Engineering, University of Montréal, Montréal, Canada
[2]Research Centre, CHU Sainte-Justine, Montréal, Canada
[3]Department of Pediatrics, Division of Neonatology, CHU Sainte-Justine and University of Montréal, Montréal, Canada
[4]Department of Radiology, Radio-oncology and Nuclear Medicine, University of Montréal, Montréal, Canada
[*]Corresponding author: Dr. Mathieu Dehaes, PhD, 3175 Côte Sainte-Catherine, Montréal, QC, Canada, H3T 1C5 (mathieu.dehaes@umontreal.ca)
[+]Equal contribution as co-senior author

ABSTRACT

Extremely preterm infants are at higher risk of pulmonary (PH) and intraventricular (IVH) haemorrhage during the transitioning physiology due to immature cardiovascular system. Monitoring of haemodynamics can detect early abnormal circulation that may lead to these complications. We described time-frequency relationships between near infrared spectroscopy (NIRS) cerebral regional haemoglobin oxygen saturation ($CrSO_2$) and preductal peripheral perfusion index (PI), capillary oxygen saturation (SpO_2) and heart rate (HR) in extremely preterm infants in the first 72h of life. Patients were sub-grouped in infants with PH and/or IVH ($N_H = 8$) and healthy controls ($N_C = 11$). Data were decomposed in wavelets allowing the analysis of localized variations of power. This approach allowed to quantify the percentage of time of significant cross-correlation, semblance, gain (transfer function) and coherence between signals. Ultra-low frequencies ($<$ 0.28mHz) were analyzed as slow and prolonged periods of impaired circulation are considered more detrimental than transient fluctuations. Cross-correlation between $CrSO_2$ and oximetry (PI, SpO_2 and HR) as well as in-phase semblance and gain between $CrSO_2$ and HR were significantly lower while anti-phase semblance between $CrSO_2$ and HR was significantly higher in PH-IVH infants compared to controls. These differences may reflect haemodynamic instability associated with cerebrovascular autoregulation and hemorrhagic complications observed during the transitioning physiology.

Supplementary Table S 1. Wavelet decomposition parameters (amplitude of the cross-correlation, semblance, gain and coherence) calculated between near infrared spectroscopy (NIRS) cerebral regional hemoglobin oxygen saturation ($CrSO_2$) and peripheral oximetry parameters, including perfusion index (PI), capillary oxygen saturation (SpO_2) and heart rate (HR). For each pair of signals, the percentage of time of significant cross-correlation (W_{xy}), semblance (S_{xy}), gain (H_{xy}) and coherence (R^2_{xy}) between any two signals were summed over the 72h period (for frequencies < 0.28mHz). Comparisons are provided between patients with a pulmonary (PH) and/or cerebral intraventricular (IVH) hemorrhage and healthy controls. General linear mixed models were used and adjusted for gestational age (GA), birth weight (BW), length of stay (LOS), pH, partial pressure of carbon dioxide ($PaCO_2$), hemoglobin concentration in the blood (HGB) and lactates.

**Variable / Independent covariate**	GA	BW	LOS	pH	$PaCO_2$	HGB	Lactates
Cross-correlation (W_{xy}) [%]							
between $CrSO_2$ and PI	**0.002**	**0.008**	**0.003**	**0.004**	**0.003**	**0.008**	**0.009**
between $CrSO_2$ and SpO_2	**$<$ 0.001**	**$<$ 0.001**	**$<$ 0.001**	**$<$ 0.001**	**$<$ 0.001**	**0.001**	**$<$ 0.001**
between $CrSO_2$ and HR	**0.020**	**0.020**	**0.015**	**0.020**	**0.007**	**0.047**	**0.032**
Anti-phase semblance ($S_{xy}\|_{\Delta\phi_{xy}=\pi\pm\pi/4}$) [%]							
between $CrSO_2$ and PI	0.434	0.381	0.377	0.250	0.308	0.443	0.169
between $CrSO_2$ and SpO_2	0.152	0.331	0.127	0.212	0.141	0.079	0.360
between $CrSO_2$ and HR	**0.041**	**0.046**	0.097	0.068	**0.045**	0.113	0.110
In-phase semblance $S_{xy}\|_{\Delta\phi_{xy}=\pm\pi/4}$) [%]							
between $CrSO_2$ and PI	0.087	0.210	0.155	**0.047**	0.085	0.115	0.057
between $CrSO_2$ and SpO_2	0.805	0.784	0.333	0.676	0.571	0.218	0.865
between $CrSO_2$ and HR	0.052	**0.034**	0.097	**0.025**	**0.026**	0.107	0.072
Gain (H_{xy})							
between $CrSO_2$ and PI	0.087	0.168	0.112	0.199	0.079	0.225	0.241
between $CrSO_2$ and SpO_2	0.695	0.645	0.519	0.747	0.598	0.864	0.806
between $CrSO_2$ and HR	0.093	0.162	0.073	0.150	0.058	0.082	0.214
Coherence (R^2_{xy}) [%]							
between $CrSO_2$ and PI	0.848	0.805	0.829	0.781	0.986	0.702	0.552
between $CrSO_2$ and SpO_2	0.376	0.375	0.745	0.786	0.566	0.809	0.504
between $CrSO_2$ and HR	0.110	0.062	0.101	**0.034**	**0.037**	0.203	0.113

p-values are generated with statistical comparisons of the means using general linear mixed models adjusted with Bonferroni correction.
